# C3-Glomerulopathy Autoantibodies Mediate Distinct Effects on Complement C3- and C5-Convertases

**DOI:** 10.3389/fimmu.2019.01030

**Published:** 2019-05-31

**Authors:** Fei Zhao, Sara Afonso, Susanne Lindner, Andrea Hartmann, Ina Löschmann, Bo Nilsson, Kristina N. Ekdahl, Lutz T. Weber, Sandra Habbig, Gesa Schalk, Michael Kirschfink, Peter F. Zipfel, Christine Skerka

**Affiliations:** ^1^Deparment of Infection Biology, Leibniz Institute for Natural Product Research and Infection Biology, Jena, Germany; ^2^Department of Immunology, Genetics and Pathology, University Uppsala, Uppsala, Sweden; ^3^Linneaus Center for Bomaterials Chemistry, Linnaeus University, Kalmar, Sweden; ^4^Children's and Adolescents' Hospital Cologne, University Hospital of Cologne, Cologne, Germany; ^5^Institute for Immunology, University Heidelberg, Heidelberg, Germany; ^6^Faculty of Life Sciences, Friedrich Schiller University Jena, Jena, Germany

**Keywords:** C3 glomerulopathy, C3NeF, C5Nef, complement, Eculizumab

## Abstract

C3 glomerulopathy (C3G) is a severe kidney disease, which is caused by defective regulation of the alternative complement pathway. Disease pathogenesis is heterogeneous and is caused by both autoimmune and genetic factors. Here we characterized IgG autoantibodies derived from 33 patients with autoimmune C3 glomerulopathy. Serum antibodies from all 33 patients as well as purified IgGs bound to the *in vitro* assembled C3-convertase. Noteworthy, two groups of antibodies were identified: group 1 with strong (12 patients) and group 2 with weak binding C3-convertase autoantibodies (22 patients). C3Nef, as evaluated in a standard C3Nef assay, was identified in serum from 19 patients, which included patients from group 1 as well as group 2. The C3-convertase binding profile was independent of C3Nef. Group 1 antibodies, but not the group 2 antibodies stabilized the C3-convertase, and protected the enzyme from dissociation by Factor H. Also, only group 1 antibodies induced C3a release. However, both group 1 and group 2 autoantibodies bound to the C5-convertase and induced C5a generation, which was inhibited by monoclonal anti-C5 antibody Eculizumab *in vitro*. In summary, group 1 antibodies are composed of C3Nef and C5Nef antibodies and likely over-activate the complement system, as seen in hemolytic assays. Group 2 antibodies show predominantly C5Nef like activities and stabilize the C5 but not the C3-convertase. Altogether, these different profiles not only reveal a heterogeneity of the autoimmune forms of C3G (MPGN), they also show that in diagnosis of C3G not all autoimmune forms are identified and thus more vigorous autoantibody testing should be performed.

## Introduction

C3 Glomerulopathy (C3G) is based on glomerular changes associated with deposition of C3 cleavage fragments and the absence of immunoglobulins (1–4). C3G represents a spectrum of related kidney disorders with autoimmune and genetic causes. A defective complement control and in particular defective fluid phase regulation of the alternative pathway (AP) C3-convertase is considered relevant for this severe kidney disorder. C3G is divided in two major subtypes, dense deposit disease (DDD), which in some cases is called membranoproliferative glomerulonephritis type II (MPGN II) and C3 glomerulonephritis (C3GN) (4–7). DDD shows specific intramembranous dense deposits and C3GN includes cases with glomerular lesions with C3 accumulation and no or little immunoglobulin staining plus mesangial, discontinuous subepithelial-, and subendothelial lesions (6, 8, 9). Immune-complex mediated MPGN is defined by glomerular IgG and C3 deposits. This form derives from the deposition of immune complexes that form in the context of infections, autoimmune diseases and malignancies which trigger the classical complement pathway (10). Multiple genetic and autoimmune causes are associated with C3G (5–7). The genetic causes include complement genes, coding for the components of the C3-convertase, C3, Factor B and for the regulator Factor H (11–17) More recently mutations in the *CFHR5* gene as well as copy number variations in the *CFHR* gene cluster were identified in C3G patients (18–24). Affected *CFHR* genes can result in hybrid FHR proteins, such as FHR1::FHR1, FHR2::FHR4, FHR2::FHR5, FHR1::FHR5, together with altered FHR plasma levels. These genetic causes in the *CFHR* gene cluster are identified in patients with MPGN I, MPGN II, DDD and C3G. In those cases with FHR hybrid proteins the disease develops in context of an intact Factor H molecule.

The diagnosis of C3G and the related disorders is primarily based on histopathology, immunohistology and identified morphological changes, C3b deposits and dense deposit formation (5–7). Defective alternative complement action either in fluid phase, in plasma or on the surface of glomerular cells and the glomerular basement membrane results in stronger C3-convertase action and in C3b deposition. Continuous C3b deposition, C3a-, C5a release, and TCC deposition ultimately results in glomerular cell proliferation and thickening of the glomerular basement membrane. In a single case of DDD the lectin pathway was associated and C4 activation and complement products were massively found in the kidney (10).

Autoimmune C3G forms with C3 Nephritic factor (C3Nef) were identified in 1969. C3Nef represent serum autoantibodies that bind to neoepitopes of the assembled alternative pathway C3-convertase, C3bBb (25–28). C3Nef does not bind to the individual components of the C3-convertase, but stabilizes the enzymatic C3-convertase (C3bBb) and extends the half-life of this central complement enzyme from a few seconds to minutes or even hours (26, 29–31). C3Nef causes continuous alternative pathway activation in plasma. In addition, to such stabilizing effects, C3Nef bound to the convertase inhibits not only the access of the inhibitor Factor H, but also of CR1 and DAF and thereby blocks the dissociation of the convertase (32, 33). As a consequence, a C3Nef-stabilized C3-convertase is continuously active in fluid phase and/or on surfaces, cleaves plasma C3 continuously, subsequenty driving complement activation. This continuous action often but not always results in C3 consumption and low C3 plasma levels, in inflammation and proliferation.

The frequency of C3Nef in C3G varies between 50 and 80%, depending on the study cohort. Variations are also influenced by age and differ between juvenile and adult patients and by the methodology used for measurement (15, 25, 34). C3Nef is also identified in patients with antiphospholipid syndrome and even in healthy individuals (35–38). In addition to C3Nef, also C4Nef and C5Nef were reported in the literature (36, 39–42). However, C3Nef assays are not standardized and the relative small number of specialized laboratories around the world use different tests.

Apparently C3Nef and properdin have related C3-convertase binding activities, and properdin binds to the assembled convertase and prolongs the half-life of the surface bound enzyme (33, 43–45). However, in contrast to C3Nef the properdin stabilized C3-convertase remains accessible for regulators and can still be dissociated by Factor H and CR1.

Recently additional autoimmune forms have been described in C3G, with autoantibodies to Factor B and C3 and for another patient with autoantibodies to Factor H. C3-convertase antibodies have been described in patients with C3G or C3G with DDD pattern (46). Importantly, the patients with these autoantibodies did not score positive in standard, functional C3Nef assays.

As autoimmune antibodies, in addition to and independent of C3Nef were reported in several C3G patients we aimed to identify and characterize these additional autoimmune forms and components in C3G and to study the effect of these autoantibodies in C3- and C5 convertase regulation. To this end, we screened the Jena C3G-registry for autoimmune C3G autoantibodies. In addition we analyzed autoantibody positive serum samples and purified IgG preparations on C3-convertase formation, stabilization and protection from the inhibitor Factor H. This approach identified 33 patients with autoantibodies, revealed differences in C3 and C5-convertase binding and action. Ca 50% of the autoantibody positive sera scored positive in standard C3Nef assays, indicating that the identification of autoimmune forms in C3G is underrepresented.

## Materials and Methods

### Patient's Samples

Sera from 33 patients (30y ±13; 12 female; 13 male) (Table 1) presenting with histological and/or clinical evidence of C3G were collected during the years 2009–2013 from clinics in Germany and Italy. The study was approved by the ethical board of the Medical Faculty of the Friedrich Schiller University, Jena Germany. In addition, sera from 7 healthy individuals were collected, pooled and used as controls or in case of the antibody screening 44 blood samples from healthy individuals were used to determine the background fluorescence in the assay. For purification of IgGs, serum was applied to a Protein G column (GE Healthcare) which was equilibrated with sodium phosphate buffer (20 mM sodium phosphate, 150 mM NaCl, pH 7.0). Bound IgGs were eluted with elution buffer (100 mM glycine-HCl, pH 2.7) and dialyzed to PBS. Control IgGs were purified from combined control sera (NHS pool). C3Nef was determined as previously described (29).

**Table 1:** Patients (*n* = 33) with autoimmune C3G.

**Patient**	**Patient code**	**Diagnose**	**Gender**	**Age at onset**	**FHRs MLPA**	**C3NeF**
**GROUP 1**						
A	#748	MPGN; I; II	f	22	CFHR1/3 het	Positive
B	#893	MPGN II	f	27	–	Positive
C	#1149	C3G	nk	nk	nd	Positive
D	#1725	C3G	nk	nk	–	Positive
E	#1742	C3G	nk	nk		Positive
F	#607	MPGN II	f	18	CFHR1/3 het	Negative
G	#1392	MPGN II	f	16	–	Negative
H	#2123	MPGN II	m	22	–	Negative
I	#2169	MPGN II	f	16	–	Negative
J	#2266	MPGN II	f	6	–	Negative
K	#2317	MPGN II	m	11	–	Negative
L	#2488	MPGN	m	15	CFHR1/3 het	Negative
**GROUP 2**						
a	#328	MPGN	f	17	nd	Positive
b	#329	MPGN	f	22	nd	Positive
c	#633	C3G	f	25	–	Positive
d	#722	C3G	m	24	3 × CFHR1/3	Positive
e	#F864	MPGN	nk	nk	nd	Positive
f	#F951	MPGN	nk	nk	–	Positive
g	#1304	IC-MPGN	nk	nk	–	Positive
h	#1549	C3G	f	13	–	Positive
i	#1710	IC-MPGN	nk	nk	–	Positive
j	#1741	C3G	nk	nk	–	Positive
k	#750	MPGN II	m	28	–	Negative
l	#971	MPGN	f	27	CFHR1/3 het	Negative
m	#2048	MPGN I	m	24	–	Negative
n	#2144	MPGN I	f	15	CFHR1/3 het	Negative
o	#2146	MPGN II	m	54	–	Negative
p	#2224	MPGNII;	f	81	–	Negative
q	#2315	MPGN	m	50	–	Negative
r	#2367	TTP; MPGN	f	15	–	Negative
s	#2390	MPGN I		40	–	Negative
t	#2461	MPGN II	m	24	CFHR1/3 het	Negative
u	#2540	C3GN	m	21	CFHR1/3 het	Negative

*Integration of patients in group1 or group 2 is based on the binding of the autoantibodies to the C3-convertase. Initial diagnosis is shown in column 3 and diseases onset, status of FHR1 and FHR3 genomic situation and scoring in the standard C3Nef assay. CFHR1/CFHR3 het, heterozygous deficiency of CFHR1 and CFHR3; –, no CFHR1/CFHR3 deficiency or multipiclation; Nd, not determine; nk, not known; MPGN, membranoprolefirative gloemerulonehpritis; C3G, C3-glomerulopathy; IC-MPGN, immune complex MPGN*.

### Autoantibody Binding to the C3 Convertase and C5 Convertase

For binding of either serum IgGs or purified patient IgGs to an *in vitro* assembled C3-convertase, C3b (5 ug/ml, CompTech) was coated onto an ELISA plate and convertases were formed by addition of Factor B (1 ug/ml, CompTech), Factor D (0.5 ug/ml, CompTech) and properdin (1 ug/ml, CompTech) in binding buffer (PBS/2% bovine serum albumin, 2 mM MgCl_2_, 2 mM NiCl_2_, 0.05% Tween 20) for 1 h at 37°C. In addition, control wells were treated with PBS. The wells were blocked and following washing patient sera (1:200 diluted in binding buffer) or purified IgGs (20 μg/ml in binding buffer) were added and the mixture was incubated at room temperature for 2 h. Upon washing bound IgGs were detected with a peroxidase-labeled anti-human IgG (1:5,500, Sigma Aldrich) and the absorbance was measured at 450 nm. Serum derived from patient # F was used as autoantibody positive sample for calculations. In order to allow comparison between the various assays, the absorbance was measured at 450 nm, and each fraction of bound IgGs was given as calculated arbitrary units (A_450_ test sample/A_450_ patient # F x 100, i.e., % binding as compared to control serum/IgGs # F. To test binding of patient IgGs to a preformed C5-convertase, ELISA plates were coated with C3b (5 ug/ml) and C5-convertases were formed by addition of Factor B (1 ug/ml), Factor D (0.5 ug/ml), properdin (1 ug/ml), and C3 (10 ug/ml, all CompTech) for 30 min at 37°C. Control wells were treated with PBS. Plates were blocked and purified patient IgGs (20 μg/ml in binding buffer see above) or patient sera (1:200 diluted in binding buffer) were added. Following incubation for 1 h at room temperature bound IgGs were detected with a peroxidase-labeled anti-human IgG (1:5,500, Sigma Aldrich). Absorbance was measured at 450 nm. C3Nef activity was measured by hemolytic assay as described (29).

### Plasma C3 and sC5b-9 Levels

C3 and sC5b9 concentrations were determined by ELISA as previously described (20).

### Alternative Pathway C3 Convertase Activity

The influence of patient IgGs on C3 convertase assembly or stability was investigated using a solid-phase C3-convertase assay. The C3-convertase, C3bBb, was assembled by coating C3b (5 μg/ml, CompTech) onto microtiter plates. Then, Factor B (1 μg/ml), Factor D (0.5 μg/ml, both CompTech), together with purified patient IgG (20 ug/ml) were added and the mixture was incubated at 37°C for 15, 30 or 45 min. Properdin (1 μg/ml, CompTech) was used as positive control. Following incubation and washing, the intensity of the formed C3 convertase was evaluated by quantitating Bb attached to the convertase with a Factor B reacting polyclonal antiserum (1:4,000, CompTech).

In addition, the stabilizing effect of the autoimmune IgGs was tested. C3-convertase was assembled in the presence of patients IgGs for 45 min. Then Factor H (20 ug/ml, CompTech) was added and the reaction was incubated at 37°C. At the indicated time points, 15, 30 or 45 min, convertase-bound Bb was detected by Factor B polyclonal antiserum (1:4,000, CompTech). The OD values were recorded at 450 nm.

### Biolayer Interferometry

The interaction of purified IgGs to the surface assembled C3-convertase was evaluated by bio-layer interferometry using a single channel BLITZ system (Forte Bio, Menlo Park, CA) and the binding affinity was determined. Ni(II)-NTA biosensors were hydrated for at least 10 min in DPBS with gelatin (0.01%) and loaded with biotinylated C3b. Then Factor B, Factor D and Properdin were added similar as described in the ELISA to assemble the C3-convertase. After washing the tip briefly (30 s) to remove unbound proteins the various IgGs were added and analyte binding was followed for 280 s. For determining the apparent kD, serial dilutions of IgGs at 2,500, 1,250, 650, 312, and 150 nM were analyzed. For each concentration, complex formation and dissociation was followed over 280 s. In addition, IgGs purified from the NHS pool and from DEAP-HUS patient #1552 who has a high titer of Factor H binding antibodies and who lacks C3-convertase reacting antibodies were used as controls. For data evaluation, values obtained from buffer controls were subtracted and the interaction affinity K_D_s were determined by fitting the data to a 1:1 model algorithm using BLITZ software. The non-linear association and dissociation curves were plotted using Graphad5 software.

### Activity of the Alternative Pathway C3-Convertase

The influence of patient IgG on C3-convertase activity was determined by measuring C3a generation. C3-convertase were assembled in the fluid phase by mixing C3b (5 ug/ml), Factor B (5 ug/ml), Factor D (1 ug/ml, all CompTech) together with purified IgGs of patients (20, 60, or 100 ug/ml) and subsequent incubation at 37°C for 8 min in MgEGTA buffer (20 mM HEPES, 144 mM NaCl, 10 mM EGTA, 7 mM MgCl_2_, pH 7.4). Then native C3 (50 ug/ml, CompTech) was added. In inhibition experiments Factor H (20 ug/ml, Comptech) was added to the mixture. The samples were incubated at 37°C, and after 40 min C3a generated in the supernatant was detected using a C3a immunoassay (QUIDEL).

### Activity of the Alternative Pathway C5-Convertase

The role of patient derived IgGs on the alternative pathway C5-convertase was monitored by following C5a generation. Purified patient derived IgGs (7.5 μg) alone or together with Eculizumab (50 nM) were added to NHS (25 μl, 10% diluted in Mg-EGTA buffer) and the mixture was incubated at 37°C. After 45 min generated C5a was detected using the C5a Plus Enzyme immunoassay (QUIDEL).

### C3b, C5b-9 Surface Deposition

Microtiter plates were coated with LPS (10 μg/ml, Sigma-Aldrich) overnight at 4°C and blocked for 1 h with BSA (2%, AppliChem Panreac). Purified patient's IgGs (10, 20, 30, or 40 μg) were added to NHS (25 μl; 20% in MgEGTA buffer) and the mixture was incubated at 37°C for 15 min. Thereafter the mixture was added to the LPS coated wells and the reaction was incubated further at 37°C for 1 h. Following washing surface deposited C3b or C5b-9 were quantified by ELISA at 450 nm using C3b mAb (1:1,000, Fitzgerald) and anti C5b-9neo (1:1,000, Comptech) and secondary anti-mouse (1:1,000, Dako) or anti-goat antiserum (1:1,000, Dako).

### Hemolytic Assay

Purified patient derived IgGs (10, 30, or 40 ug) were added to NHS (10%) and the mixtures incubated for 20 min at 37°C with agitation (550 rpm). Rabbit erythrocytes (2^*^10^8^/ml) were washed, suspended in GVB^**^ buffer (Comptech), added to IgGs-NHS and the mixture was then incubated for additional 30 min at 37°C with agitation (450 rpm). Cells were centrifuged and erythrocyte lysis was evaluated by measuring the absorbance in supernatant at 414 nm.

### Statistical Analysis

Significant differences between two groups were analyzed using the unpaired two tailed Student's *t*-test of GraphPad Prism 5 for Windows. Values of ^*^p ≤ 0.05, ^**^p ≤ 0.01, ^***^p ≤ 0.001 were considered as statistically significant.

## Results

### Identification of C3G Patients With Autoantibodies

**Figure 1:**
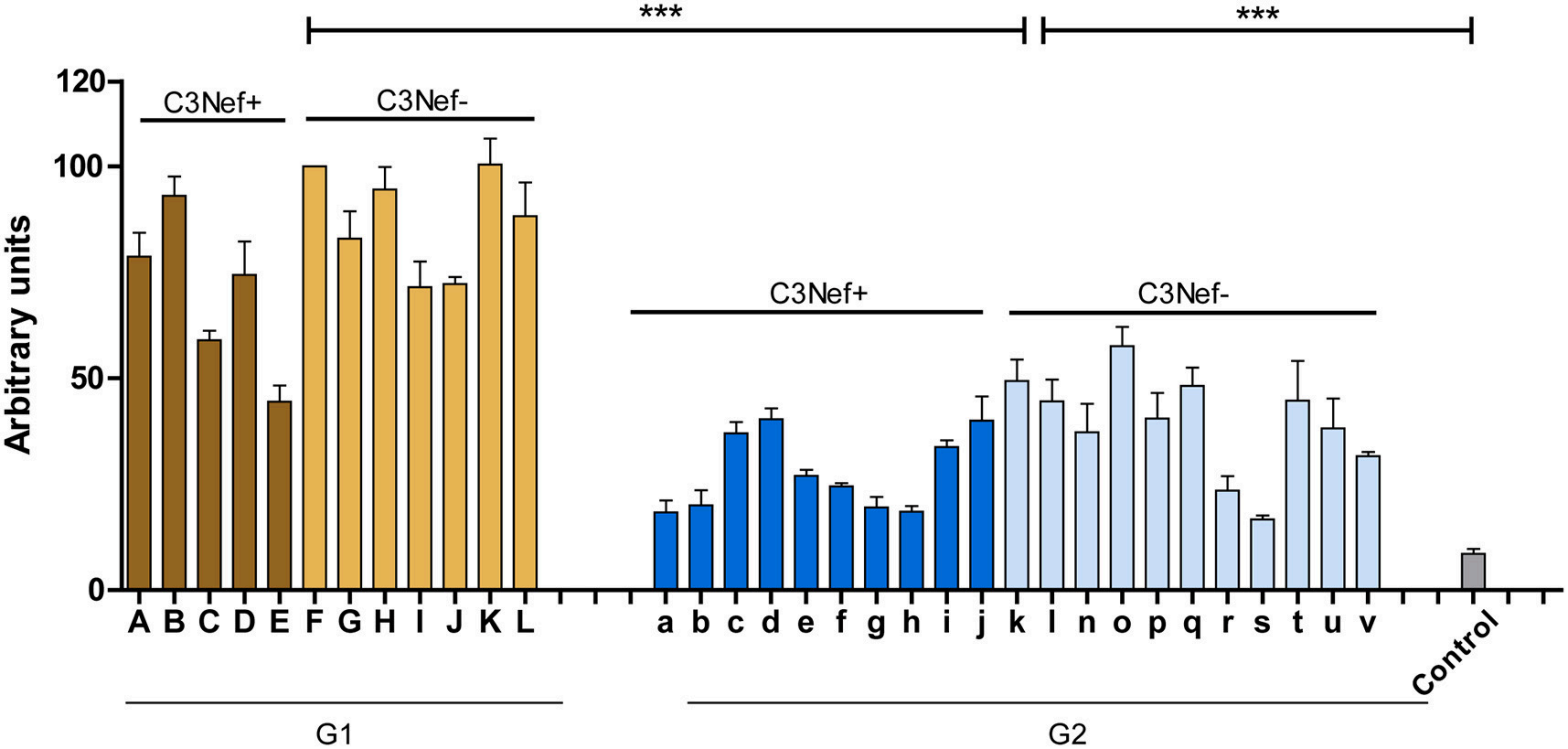
C3G patients presenting with autoantibodies to the C3-convertase. Thirty-three autoimmune patients with C3G with autoantibodies that bind to the *in vitro* assembled C3 convertase were identified in the Jena C3G cohort. Based on the binding intensity of autoantibodies to the *in vitro* assembled C3-convertase patients with high and low binding intensities are identified. Separated in group 1 with high/more C3-convertase binding antibodies and group 2 with weak/less binding antibodies. Each group includes patients who score positive in a C3Nef assay as well as C3Nef negative patients. Cut off was set at arbitrary units of 8.5 (average background binding level of 44 control sera). G1 to G2 and G2 to controls (***p < 0.001).

To identify and characterize autoimmune C3G patients in the European/Jena cohort in more detail, serum samples of 250 nephritis patients were analyzed for autoantibodies which bind to the *in vitro* assembled alternative pathway C3-convertase. Thirty-three C3G patients were identified having C3bBb binding antibodies. Twelve sera showed strong binding (arbitrary units: 80 ± 4.8) and 21 patients showed weak binding (arbitrary units: 34 ± 2.6) as compared to IgGs from 44 NHS control samples (arbitrary units: 8.5 ± 1.2, *p* < 0.001) (Figure 1). Based on the different binding intensities two groups were separated, patient with high binding-, termed group 1 and patients with low binding antibodies, as group 2. The demographic data of the patients in these subgroups were rather homogenous for age and gender (Table 1).

All 33 sera were further analyzed using a standard assay for C3 Nephritic factor. Fifteen sera (42%) were positive for C3Nef which were identified in both group 1 and group 2 patients. Thus, in group 1 sera from seven individuals were negative and five sera were positive for C3Nef. Among group 2, 11 sera were negative and 10 were positive for C3Nef (Figure 1 and Table 1).

In DEAP-HUS the presence of Factor H antibodies is associated with homozygous *CFHR1-CFHR3* deficiency. Copy number variations in the *CFHR* gene cluster were determined for patients with C3-convertase antibodies. In particular no homozygous *CFHR3/CFHR1* deficiency was detected; seven patients showed heterozygous *CFHR3-CFHR1* deficiency (Table 1). Thus, autoimmune antibodies in C3G patients develop independently of *CFHR3/CFHR1* homozygous deficiency.

### Estimating Binding Affinity of Autoantibodies

All here tested autoantibodies bound to the assembled C3-convertase, but with different intensity. These differences can be explained either by higher autoantibody titers in group 1 sera, or by a higher binding affinity to the C3-convertase. To find out if the autoantibodies bind stronger or weaker, binding of representative IgG preparations of group 1 or group 2 with a C3Nef negative and C3Nef positive variant of the C3-convertase was followed in real time and the apparent affinity was evaluated. The C3-convertase was assembled on the surface of a Ni(II)-NTA coated biosensor and purified IgGs from group 1 patients (#A C3Nef pos and #J C3Nef neg) of group 2 patients (#a C3Nef pos and #r C3Nef neg) were added as analyte. Association and dissociation was followed for 320 s. IgG fractions isolated from the patients harbored autoantibodies that bound to the assembled convertase. They showed a strong association and a slow dissociation upon removal of the analyte. In contrast, IgGs isolated from the NHS pool, from DEAP-HUS patient #1552, and also BSA did not bind to the C3 convertase. To compare the binding strength and to define an apparent K_D_, serial dilutions ranging from 25 to 75 nM were tested. Assuming that 10% of total IgG isolation represent C3-convertase binding autoantibodies the apparent K_D_ values were calculated and identified for group 1 as 911 and 853 nM and for group 2 as 416 and 559 nM (Table 2). Control IgGs prepared from the NHS pool or from DEAP-HUS patient #1552 showed no, or background binding (K_D_ > 2.5 μM). The results demonstrate that autoantibodies from representative patients from both groups bind with rather similar affinity and binding under the assumption of 10% C3 convertase antibodies is in the nanomolar range. The differences in binding could be explained by the recognition of different epitopes.

**Table 2:** Binding of selected purified antibodies to the AP C3-convertase.

	**C3NeF**	**Patient**	**K_**D**_ (nM)**	**k_**a**_ (nM)**	**k_**d**_ (1/s)**
Group 1	pos	#A 748	416	4.8	2.0 × 10^−3^
	neg	#J 2266	911	2.3	2.1 × 10^−3^
Group 2	pos	#a 328	559	4.5	2.5 × 10^−3^
	neg	#r 2315	854	4.5	3.8 × 10^−3^
NHS	neg		2,513	1.9	5.0 × 10^−3^
DEAP-HUS	neg	#1552	3,799	1.4	5.4 × 10^−3^

### Group 1 but Not Group 2 Autoantibodies Enhance C3-Convertase Formation and Block Dissociation by Factor H

**Figure 2:**
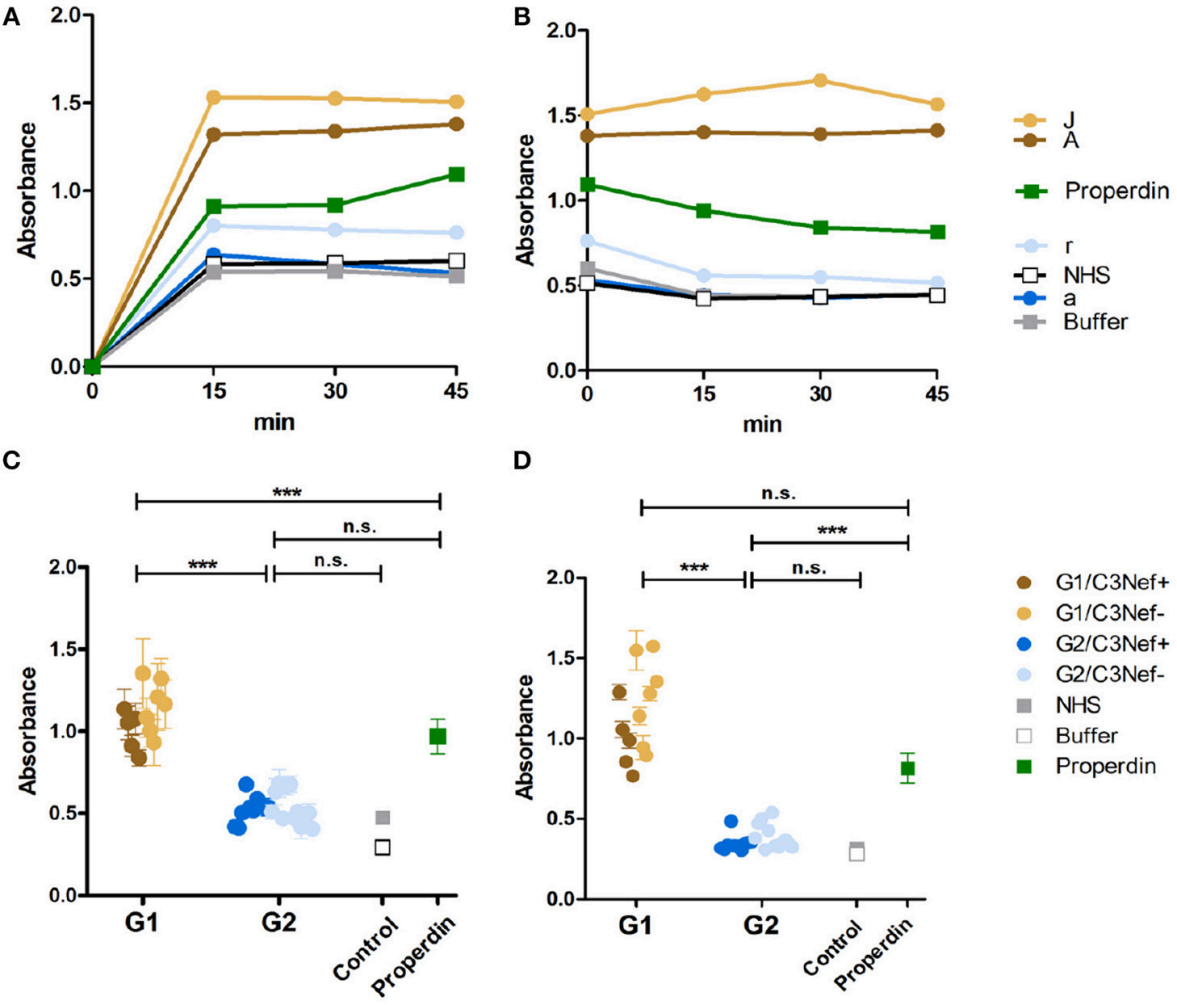
C3G autoantibodies of group 1 and group 2 influence C3 convertase assembly and dissociation by Factor H. **(A)** Two group 1 autoantibodies from patients #A and #J with high C3 convertase binding IgGs enhance C3-convertase formation. This enhancing effect is even stronger than the C3 convertase stabilizer and complement activator properdin. In contrast, group 2 autoantibodies from patients #a and# r do not enhance C3 convertase formation. Their effect is comparable to IgGs derived from NHS or from a DEAP-HUS patient with autoantibodies to Factor H or to buffer. **(B)** The same group1 antibodies when bound to the 3 convertase stabilize the enzyme and enhances resistance to factor H mediated dissociation. C3-convertase assembly *in vitro* and stabilization by autoantibodies are measured over 45 min by detection of convertase Bb using ELISA. **(C)** The same experiments as outlined in panel **(A)** are performed with all 33 patients derived autoantibody fractions and bound IgGs are identified after 45 min. All 12 group 1 antibodies enhance C3-convertase formation and again all group 2 antibodies have no or rather low effects (***p < 0.001). **(D)** All group 1 autoantibodies stabilize the C3 convertase from dissociation by factor H and all group 2 antibodies lack this activity (***p < 0.001).

To analyze whether all C3-convertase binding autoantibodies have a stabilizing and activating effect we analyzed if the autoantibodies influence C3-convertase formation. First, purified IgGs from representative sera of group 1 (#A and #J) and group 2 (#a and #r) patients were tested in a functional kinetic assay. Again, the C3-convertases were assembled in presence of purified IgGs and convertase formation was followed by measuring attached Bb. IgGs from the two group 1 patients (#A and #J) enhanced C3-convertase assembly within the first 15 min and over time more C3-convertases were formed. The effect was more pronounced as that of properdin, the known C3-convertase stabilizer. In contrast, IgGs from the two group 2 patients (#a and #r) did not affect convertase formation in a significant manner. Their effect was lower than that of properdin and was almost comparable to NHS or buffer control (Figure 2A). Thus, group 1 IgGs allow faster C3-convertase formation and the formation of more enzyme (Figure 2A).

Next we asked if the IgG autoantibodies influence convertase dissociation by the inhibitor Factor H. To this end, C3-convertases were assembled in presence of the autoantibodies and Factor H. In this setting both group 1 autoantibodies (#A and #J) inhibited convertase dissociation by Factor H and the stabilizing effect on the assembled C3-convertase was even stronger than that of properdin (Figure 2B). In contrast, both group 2 antibodies (#a and #r) did not or weakly block C3-convertase dissociation by factor H (Figure 2B).

To evaluate if these differences are characteristic for the two groups, all IgGs were evaluated. All twelve group 1 autoantibodies efficiently stabilized the C3-convertase as shown by Bb binding (mean absorbance group 1: 1.1, *p* < 0.001, compared to NHS pool) (Figure 2C) even in the presence of Factor H (Figure 2D). All group 2 autoantibodies, had a rather low or no stabilizing effect on the C3-convertase (mean absorbance group 2: 0.5, n.s. difference to NHS pool) (Figure 2C) and no major influence on Factor H stabilization (Figure 2D). The effect by group 2 autoantibodies was comparable to IgGs derived from the control NHS pool or buffer. In summary, group 1 but not group 2 autoantibodies increased C3-convertase formation and stabilized the enzyme from decay by Factor H. A significant difference exists between group 1 and group 2 antibodies. IgGs from group 1, but not the group 2 group stabilized the C3-convertase. In each group, no significant difference was detectable between the C3Nef positive or the C3Nef negative samples. The effect of all group 1 autoantibodies was stronger than that of properdin, the known C3-convertase stabilizing protein and complement enhancer. This may explain the pathologic role of the antibodies.

### Effect of Autoantibodies on C3-Convertase Activity

**Figure 3:**
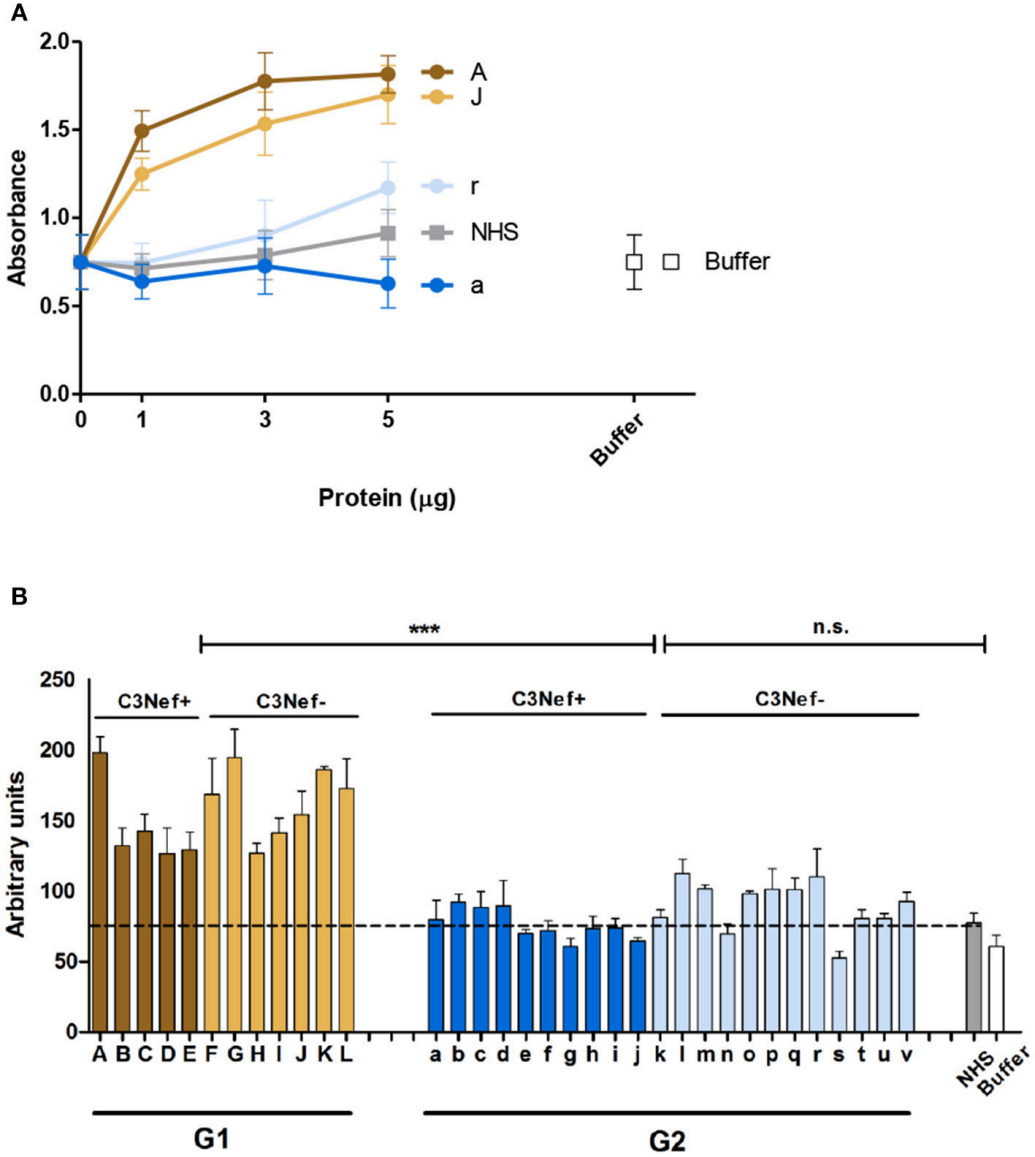
Group 1 but not group 2 antibodies enhance C3a generation. Fluid phase C3-convertase is assembled in presence of autoantibodies followed by addition of substrate C3. Following incubation for 45 min, C3a generation is monitored by ELISA. **(A)** Group 1 autoantibodies increase C3a generation and the effect is dose dependent. In contrast, group 2 antibodies do not enhance C3a formation. **(B)** When all autoantibodies were evaluated group 1 but not group 2 autoantibodies enhance C3a generation (***p < 0.001). C3a generation by NHS is shown as a stippled line.

As group 1 autoantibodies enhanced C3-convertase formation, stabilized the enzyme and blocked the dissociation, we asked if the IgG autoantibodies influence C3-convertase activity. Therefore, C3-convertases were assembled in fluid phase in presence of the autoimmune IgGs, and after 30 min incubation generated C3a was quantitated by ELISA. The selected group 1 antibodies (i.e., #A and #J), but not the two group 2 antibodies (#a and #r) enhanced C3a generation, and the effect was dose dependent (Figure 3A). IgGs from C3Nef negative patient #r, had some activating activity at higher concentrations, but IgGs prepared from the C3Nef pos patient #a lacked any activity (Figure 3A).

Similar results were obtained when IgGs from all patients were analyzed. All high binding C3-convertase group 1-IgGs enhanced C3a formation (mean absorbance G1: 156, mean absorbance G2: 84; *p* < 0.0001 Group 1 compared to group 2). The group 2 IgGs had minor or no effects on C3a release (*p* = n.s. compared to NHS pool) (Figure 3B). Again, no difference between C3Nef positive and C3Nef negative samples was detectable. Thus, the high binding group 1 antibodies enhanced C3-convertase action also in the fluid phase which supports their role in pathology even further. Identified C3Nef in group 1 but not C3Nef in group 2 did activate the C3-convertase. These results are in agreement with C3 concentrations in patients' samples. G1 samples showed very low C3 amounts (103 ± 17 μg/ml) compared to G2 samples (331 ± 80 μg/ml; *p* < 0.0001).

### Both Group 1 and Group 2 Autoantibodies Bind to the C5-Convertase

**Figure 4:**
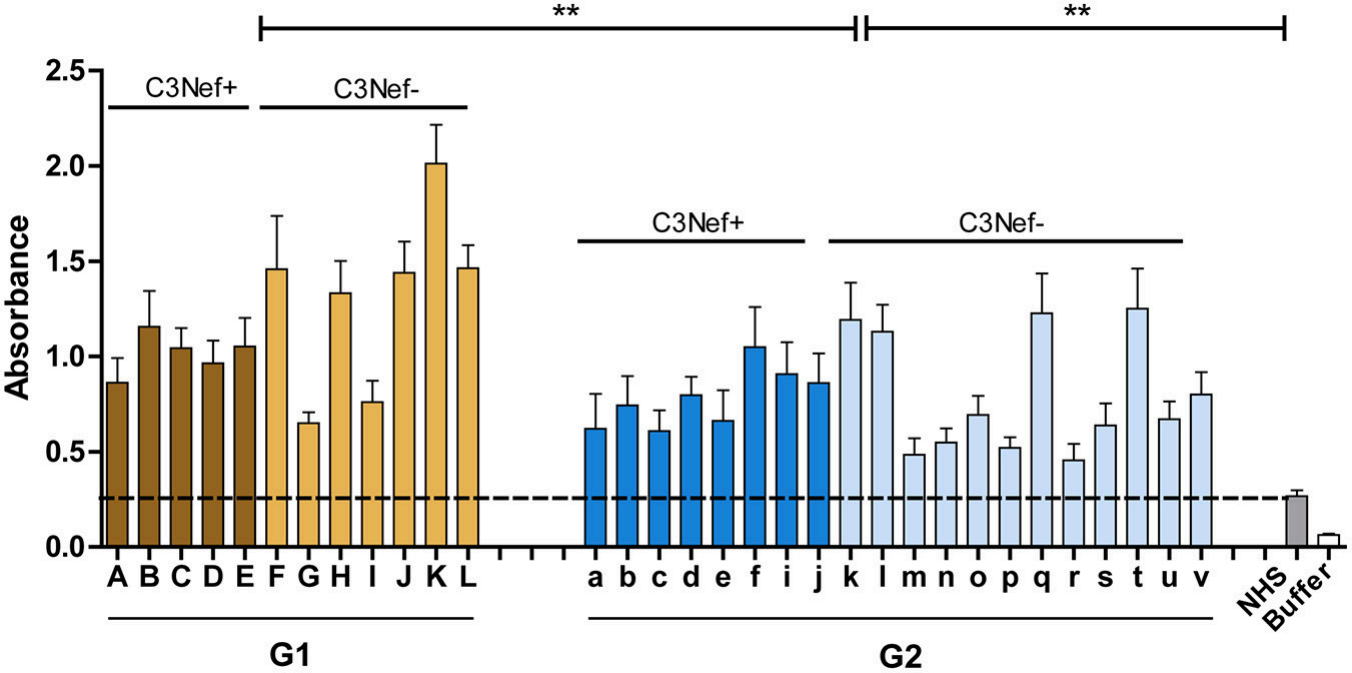
C5 Convertase binding. Autoantibody binding to the *in vitro* assembled C3 convertase was evaluated. In this case, group 1 and also group 2 antibodies bind to the assembled C5-convertase (**p < 0.01). IgGs prepared from NHS show background binding (stippled line). In addition, more group 1 antibodies bind to the C5-convertase as compared to group 2 antibodies (**p < 0.01). The C5-convertase was assembled on an ELISA plate by immobilizing C3b (10 μg/ml) and adding Factor B (1 μg/ml), Factor D (0.5 μg/ml), properdin (1 μg/ml), and C3 (10 μg/ml).

Group 1 and group 2 antibodies both bind to neoepitopes of the C3-convertase. As the AP C5-convertase, C3bBbC3b, with one additional C3b attached has a related structure or composition, we asked if the autoantibodies also bind to this enzyme. Therefore, autoantibody binding to AP C5-convertases assembled on the surface of a microtiter plate was evaluated by ELISA. Autoantibodies derived from all patients of the cohort bound to the assembled C5-convertase. Also for C5-convertase interaction, the high C3-convertase binding group 1-autoantibodies bound also more to the alternative pathway C5-convertase than group 2 antibodies (mean absorbance group 1: 1.2; mean absorbance group 2: 0.8; *p* < 0.01 group 1 compared to group 2) (Figure 4). In this set up both group 1 and group 2 antibodies bound with significantly higher intensity to the C5-convertase as NHS derived IgGs (*p* < 0.01).

### Most Autoantibodies Activate the C5-Convertase

**Figure 5:**
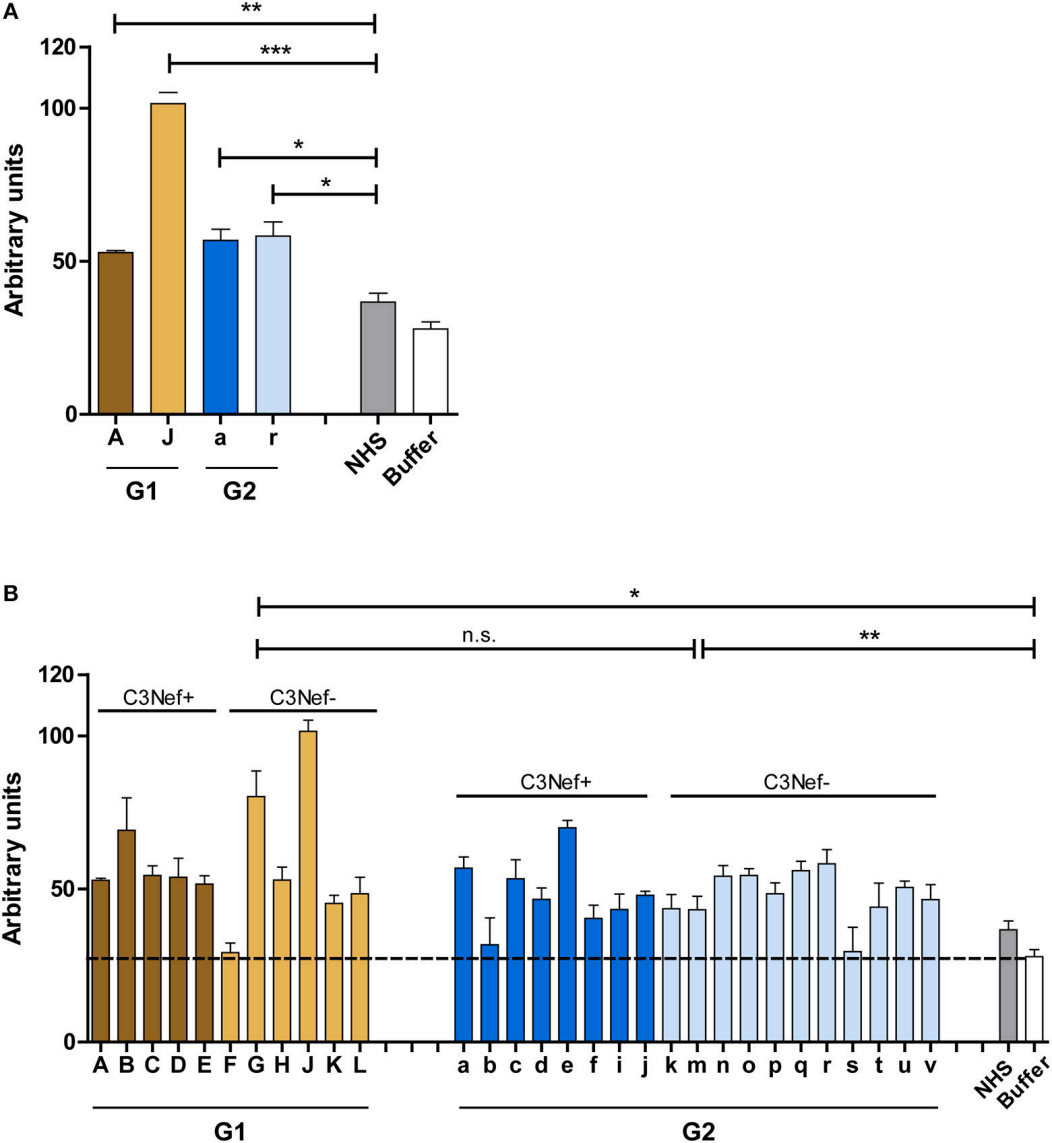
Autoantibodies modulate C5a generation. **(A)** Representative patient's autoantibodies from group 1 (#A and #J) and group 2 (#a and #r) increase C5a generation significantly when compared to IgGs prepared from NHS compared to control (**p < 0.01, ***p < 0.001). **(B)** IgGs from all patients except # 607 (group 1), # 328, #2367 (both group 2), enhance C5a generation in a significant manner (*p < 0.05, **p < 0.01, ***p < 0.001). Purified IgGs from each patient was added to complement active NHS at 37°C and the mixture was incubated for 45 min. C5a generation was monitored by ELISA and compared to C5a generation in NHS (stippled line).

Based on binding to the C5-convertase we asked if the autoantibodies affect C5-convertase activity. First, IgGs from the four selected patients were added to NHS and after 45 min incubation newly generated C5a was determined in the supernatant by ELISA. All four antibody IgG preparations (#A, #J, #a, and #r) increased C5a generation (*p* < 0.05 to 0.001 as compared to NHS-IgGs) (Figure 5A). When IgGs from all patients were tested all, except one sample (# F) from group 1 and two samples from group 2 (#b and #s) increased C5a generation. The IgGs from both groups generated rather similar C5a levels, however, the range was higher for group 1 autoantibodies (mean absorbance group 1: 58, mean absorbance group 2: 48, G1 to G2 *p* = n.s.; G1 to buffer *p* < 0.05; G2 to buffer *p* < 0.005) (Figure 5B). Again, this effect was independent and did not correlate with the presence or absence of C3Nef.

### IgG Antibodies Induce C3b Deposition

**Figure 6:**
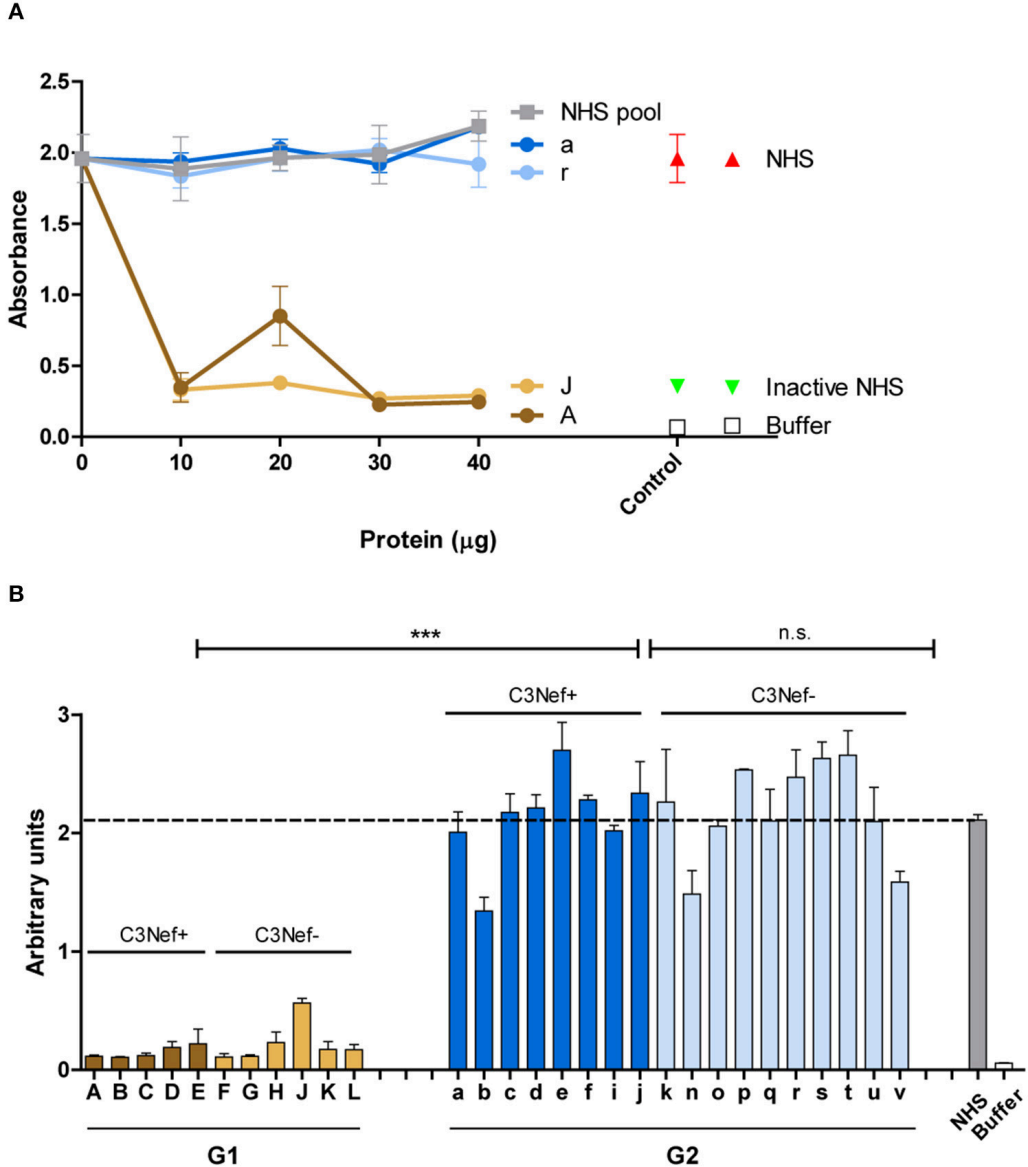
Group 1 but not group 2 antibodies affect surface C3b deposition. **(A)** Two representative group 1 autoantibodies (#J and #A) inhibit C3b deposition and the effect is dose dependent. In contrast, both group 2 antibodies (#a and #r) fail to reduce C3b deposition. C3b deposition was followed with adding increasing amounts of IgGs (10–40 μg) to complement active NHS and after surface deposited C3b was quantitated by ELISA **(B)** Autoantibodies (each 30 μg) from all patients were tested in the same set up. Again, all group 1, but not group 2 antibodies block C3b surface deposition (***p < 0.001). Purified IgG in **(A,B)** were added to NHS, and the supplemented serum was added to a LPS-coated microtiter plate. After 1 h C3b deposition was assayed by ELISA. Hemolysis by NHS is marked by a stippled line.

Next the effect of the autoantibodies on fluid phase C3-convertase mediated C3b opsonization was evaluated. Purified patients IgGs were added to NHS and after 30 min surface deposited C3b was quantitated by ELISA. Both group 1 antibodies (#A and #J) reduced C3b surface deposition and this inhibitory effect was already detected with 10 μg/ml IgG (Figure 6A). In contrast, both group 2 antibodies (*#a* and #r), as well as NHS-derived IgGs did not affect C3b deposition (Figure 6A).

The inhibitory effects were specific for group 1. All group 1 antibodies, but no group 2 antibody blocked C3b deposition (mean absorbance G1: 0.2, mean absorbance G2: 2.2; *p* < 0.001 group 1 vs. group 2) (Figure 6B). These results reveal a functional difference between group 1- and group 2 autoantibodies in C3b deposition.

### Autoantibodies Influence TCC Deposition

**Figure 7:**
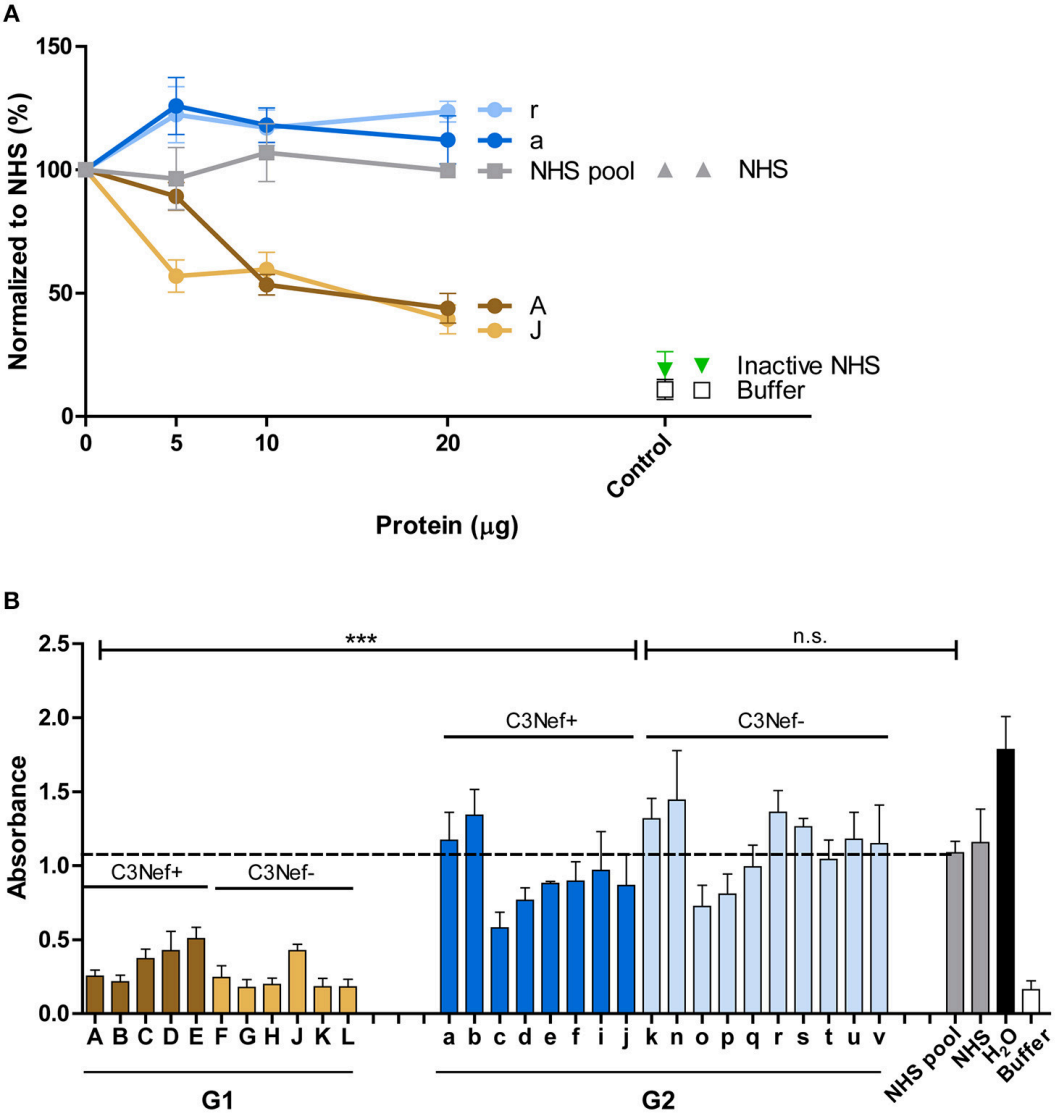
Group 1 but not group 2-antibodies reduce C5b-9 surface deposition and prevent hemolysis. **(A)** Group 1 autoantibodies (#J and #A) inhibit C5b-9 deposition by about 50% and the effect is dose dependent. In contrast, group 2 antibodies (#a and #r) enhance C5b-9 deposition as compared to NHS derived IgGs. C5-9 deposition was followed upon addition of increasing amounts of IgG fractions (5–20 μg) to NHS. **(B)** Autoantibody fractions derived from group 1 patients (each 30 μg) inhibit lysis of rabbit erythrocytes (***p < 0.001). Purified IgGs were added to NHS, then this mixture was combined with rabbit erythrocytes. Hemolysis of erythrocytes was assayed by measuring the absorbance. Lysis of erythrocytes in NHS is marked as stipled line.

The effect of the autoantibodies on TCC formation was evaluated by following C5b-9 deposition and using a hemolytic assay. First, the representative antibodies from group 1 (#A and #J) and group 2 (#a and #r) were evaluated. The IgGs were added to NHS and the deposition of C5b-9 was measured by ELISA. IgGs derived from the group patients (#A and #J) decreased C5b-9 deposition in a dose dependent manner, whereas group 2 autoantibodies (#a and #r) enhanced C5b-9 deposition when compared to NHS derived IgGs (Figure 7A). sC5b-9 concentration in the samples ranged from 3.4 ±1.3 μg/ml in group 1 to 7.4 ± 2.3 μg/ml in group 2.

The differences between group 1 and group 2 antibodies was confirmed for all autoantibodies using a hemolysis assay. The IgGs derived from the patients were added to NHS and then rabbit erythrocytes which represent activators of human complement were challenged with the supplemented serum and after 20 min incubation lysis of erythrocytes was followed. All group 1, but no group 2 antibody blocked hemolysis (mean absorbance group 1: 0.3, mean absorbance group 2: 1.0, *p* < 0.001 group 1 compared to group 2; *p* = not significant group 2 to NHS pool) (Figure 7B). This clear difference between group I and group II antibodies was again independent of the presence or absence of C3Nef. Thus, a clear functional difference between group 1 and group 2 antibodies in autoimmune C3G affects complement mediated TCC deposition and hemolysis.

### Eculizumab Inhibits C5a Generation in All Probes

**Figure 8:**
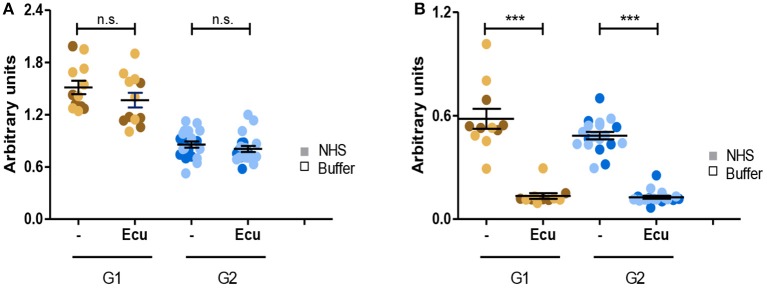
C5 inhibitor Eculizumab reduces C5a but not C3a generation of convertases with autoantibodies attached. **(A)** Eculizumab does not affect C3a generation. Fluid phase C3 convertases were assembled in presence of group 1 or group 2 antibodies and C3a release was monitored by ELISA. In the absence of Eculizumab group 1 but not group 2 antibodies induced C3a release (panels 1, 3). C3a generation was not enhanced in group 2 (panels 2, 4). **(B)** Eculizumab blocks C5a generation of C5-convertases with either group 1 or group 2 antibodies attached (***p < 0.001). Eculizumab was added to NHS together with group 1 or group 2 antibodies (each 30 μg). Following incubation C5a generation was measured by ELISA.

Given that all C3G autoantibodies activate the C5-convertase we asked whether the C3G autoantibodies influence the action of Eculizumab, the therapeutic C5 binding complement inhibitor. Fluid phase C3-convertases were assembled in presence of autoantibodies or in presence of autoantibodies and Eculizumab. After addition of C3 to the mixture and 45 min incubation, C3a as well as C5a, generated in fluid phase, was quantitated by ELISA. As expected, Eculizumab did not influence C3a release (Figure 8A). However, Eculizumab blocked C5a release in presence of both group 1 as well as group 2 autoantibodies (Figure 8B). Thus, C5 binding Eculizumab blocks the action of a C5-convertase which has the C3G autoantibody attached. Eculizumab and autoantibodies bind to different proteins. Eculizumab binds to the substrate C5 and the autoantibodies bind to different neoepitopes of the C5-convertase.

## Discussion

Here we identified and characterized autoimmune forms of the kidney disease C3G. A cohort of 33 C3G patients displayed autoantibodies which bound to both the C3- and the C5-convertase of complement. All autoantibody isolates activated the C5-convertase (C5Nef), but only half of them activated in addition the C3-convertase (C3Nef). IgG fractions with C3Nef plus C5Nef (group 1 antibodies) had stronger complement activating functions in *in vitro* assays compared to fractions with C3Nef alone (group 2 antibodies). Group 1 antibodies induced a fast consumption of the complement components C3 and C5 in hemolytic assays. Eculizumab, the therapeutical C5 monoclonal antibody inhibits C5 cleavage and C5a generation triggered by each group 1 or group 2 antibody fraction, but as expected has no effect on enhanced C3 cleavage.

About 45% of all autoantibodies in our cohort were identified as C3Nef using the classical hemolytic assay. This assay identifies autoantibodies that bind to a neoepitope formed in the C3-convertase. However, additional autoimmune antibodies than C3Nef add to C3G, which are not identified by the classical C3Nef assay. These antibodies bind to the assembled C3-convertase or components thereof. In addition, several C3Nef antibodies bound to the C3-convertase but did not lead to the activation of the enzyme *in vitro*. In contrast, initially identified C3Nef antibodies bound/activated the C5-convertase.

Based on the reactivity to the assembled C3-convertase, two groups of autoantibodies were identified. Group 1 and group 2 antibodies, which both had high affinities in binding to the assembled C3-convertase, but exclusively group 1 antibodies also activated the C3-convertase. As the binding affinities of IgGs to the C3-convertase were very similar in both groups, the different functional effects between the groups are explained by either a higher titer of the autoantibodies in each isolated IgG fraction of group 1 patients or by a higher C3-convertase stabilizing effect by group 1 antibodies compared to group 2. Different antibody titers between the two groups were excluded, as dilutions of group 1 antibody preparations did neither influence nor diminish the stabilizing effect or the resistance to convertase decay by Factor H. Thus, the functional differences between group 1 and group 2 antibodies are likely independent of the autoantibody titers and rely on the recognition of different epitopes which are recognized in the C3- and C5-convertases. All autoantibody fractions from the patients bound to the C5-convertase and as most of them enhanced the activity, they are likely similar to previously defined C5Nef (46). This is surprising as group 1 and group 2 autoantibodies contained C3Nef (according the C3Nef assay) which stabilize the C3-convertase but not the C5-convertase. Therefore, we conclude that two different types of antibody carriers were identified in this cohort of C3G patients: C3Nef+/C5Nef+ (stabilizing the C3-convertase and also the C5-convertase) and C3Nef–/C5Nef+ (stabilizing the C5 convertase) patients. A similar classification was found in a cohort study described by Donadelli et al. (47), who in addition identified patients with exclusively C3Nef antibodies. Thus, C3G patients are heterogeneous and include patients with C3Nef or C5Nef or combinations thereof. This heterogeneity results in presence of nephritic factors with different functional activities and consequences *in vivo* for the patient. In the Jena cohort, nearly all patients were C5Nef positive and show that a large and substantial fraction of autoimmune C3G patients are not identified by only a standard C3Nef assay.

Genetic copy number analyses of the *CFHR* gene cluster revealed for all 33 autoimmune C3G patients no homozygous deletion in the *CFHR gene* cluster. This lack of correlation with CFHR copy number variations is in clear contrast to DEAP-HUS patients, i.e., patients who present with the autoimmune form of hemolytic uremic syndrome. DEAP-HUS patients who developed autoantibodies to Factor H present frequently with a homozygous deletion of the *CFHR1-CFHR3* segment or alternatively but rarely by a compound heterozygous *CFHR1-CFHR3*, together with *CFHR3-CFHR4* deletions (48).

There is no clear therapy regiment for C3G today. As serum derived autoantibodies, as well as purified IgGs in our cohort bound to the *in vitro* assembled C5-convertase and enhanced fluid phase C5a generation, the question arose whether inhibition of C5 by Eculizumab is a therapeutic option. Off-label use of Eculizumab in C3G revealed promising results in some, but not all patients (49–51). *In vitro* assays in our cohort confirmed inhibition of C5 by Eculizumab and reduced hemolysis. However, the inhibitory effect was exclusively present in group 2, with C5Nef only. In group G1 carriers with C3Nef plus C5Nef, complement activation is likely too strong to be restricted by Eculizumab alone. Whether these patients benefit or profit from a stratified immunosuppressive therapy needs to be determined in the future in clinical practice.

## Ethics Statement

The study was approved by the ethics committee of the University Hospital of Jena, Germany.

## Author Contributions

FZ, MK, SA, SL, IL, and AH performed experiments and analyzed the data. BN and KE helped in C3Nef assays. LW, SH, and GS provided patient's samples. All authors read the manuscript and discussed the data. PZ and CS designed the study, analyzed data, and wrote the manuscript.

### Conflict of Interest Statement

The authors declare that the research was conducted in the absence of any commercial or financial relationships that could be construed as a potential conflict of interest.
